# The Role of Israel’s Emergency Medical Services During a Pandemic in the Pre-Exposure Period

**DOI:** 10.1017/dmp.2020.369

**Published:** 2020-10-12

**Authors:** Eli Jaffe, Roman Sonkin, Timna Podolsky, Evan Avraham Alpert, Maya Siman-Tov

**Affiliations:** Magen David Adom, Tel Aviv, Israel; Shaare Zedek Medical Center, Jerusalem, Israel; Tel Aviv University School of Public Health, Tel-Aviv, Israel

**Keywords:** coronavirus, COVID-19, emergency medical services, epidemics, pandemics

## Abstract

**Objective:**

The scientific literature on coronavirus disease (COVID-19) is extensive, but little is written about the role of emergency medical services (EMS). The objective of this study is to describe the role of Magen David Adom (MDA), Israel’s national emergency prehospital medical organization, in the pre-exposure period, before widespread governmental action. These efforts were based on (1) phone diagnosis, dispatch, and transport; and (2) border management checkpoints.

**Methods:**

This is a descriptive study of MDA’s role in pandemic response during the pre-exposure period. Medical emergency telephone calls from either individuals or medical sources were identified by a dispatcher as “suspected COVID-19” based on symptoms and travel exposure. Data were also collected for travelers approaching the MDA border checkpoint at Ben-Gurion International Airport.

**Results:**

The total number of protected transports during this time was 121. Of these, 44 (36.3%) were referred by medical sources, and 77 (63.7%) were identified as “suspected COVID-19” by dispatchers. The checkpoint was accessed by 156 travelers: 87 were sent to home-quarantine; 12 were transported to the hospital; 18 were refused entry; and 39 required no further action.

**Conclusion:**

EMS can work effectively in the pre-exposure period through instructing home quarantine, providing protected transport, and staffing border control checkpoints.

Pandemics break out due to transference of infection between individuals, involving uncontrolled mass exposure and global transmission. Attempts to increase public health protection create a surge in the consumption of medical services.^1,2^ At the most critical level, this includes the need for more intensive care unit beds and ventilators. Demands from staff and material resources increase and coordination is required among many health agencies.^2^ Pandemics also drive a heavy consumption of medical resources, including those of emergency medical services (EMS).^3^

The process of infection on a global scale follows a timeline that calls for varying degrees of alertness and preparedness as the disease spreads. The World Health Organization (WHO) identifies 6 stages of pandemic preparedness and response. After community-level outbreaks have been verified (WHO, stage 4), medical professionals must attempt rapid containment, manage multiple resources, and continue updating the public.^4^ The Centers for Disease Control and Prevention (CDC) identifies 7 domains of action in the response of medical services to pandemic risks. In the pre-outbreak stage, the first domain is *Surveillance, Epidemiology, and Laboratory Activities*.^5^ A 2012 study called for increasing the role of EMS in pandemics and particularly in ongoing disease surveillance and mitigation strategies.^6^ Besides being responsible for the traditional role of stabilizing and treating patients in emergent prehospital situations and transporting them to definitive care, EMS can provide the first layer of rapid recognition and response before the implementation of pandemic protocols. During pandemics, the role of EMS in managing risk is a front-line position.^7^

EMS organizations are not only active during an infectious outbreak, but also in the pre-exposure period when the global pandemic is spreading but has not yet been identified in that specific region. However, a study from the United States claims that most EMS agencies are fundamentally unprepared for a pandemic event.^8^

Border management plays a debated role in early containment efforts. According to the CDC Pandemic Influenza Plan, the goal is to detect those suspected of infection at the border so that they can be placed in isolation or prevented from entering the country and spreading the disease.^5^ The WHO 2015 guidance on pandemics suggests considering entry screening in countries not yet affected.^9^

Based on previous pandemics, Israel’s Ministry of Health (MOH) chose to assemble a full preparedness plan, so that in 2009 when H1N1 broke out globally, the country was well prepared with protocols for hospitals, community health care, and prehospital services.^10^ Magen David Adom (MDA), Israel’s national emergency prehospital medical organization, is accustomed to operating in a continuous state of flexibility due to its experience in mixed civilian/military scenarios based on many years of operating in a heavily conflicted region.^11^

As the national EMS organization, MDA must balance the increased needs created by a pandemic risk with the ongoing national EMS needs. When dealing with large-scale events such as a mass casualty incident or pandemic, MDA designates resources based on projection rather than on immediate need, thereby increasing the preparedness factor.^12^ Preparedness for pandemics must begin in the pre-exposure stage so that containment can be at the highest possible level and personal protective equipment can be worn by EMS in anticipation of the earliest detection.

MDA responds to thousands of calls daily (via the designated medical emergency number 1-0-1) through 8 regional dispatch centers staffed solely by paramedics and emergency medical technicians (EMTs). In both standard and large-scale events, MDA operates as the primary source of triage, dispatch, treatment, and transport. MDA operates a sophisticated technological command and control platform that allows rapid intake, telephone guidance, auditing, archiving, automatic ambulance dispatch, and automatic mobilization of volunteer first responders using a designated smartphone application. The application allows access to pre-uploaded medical information, transmitting Global Positioning System coordinates, and video calling. The technology enables rapid live updates and consultations about cases with medical specialists both internal and external to MDA. Databases are available to the relevant governmental agencies.

## EMS PRE-EXPOSURE MANAGEMENT OF COVID-19 IN ISRAEL

During January 2020, the novel coronavirus, severe acute respiratory syndrome coronavirus 2 (SARS-CoV-2), which was the causative agent of the epidemic pneumonia outbreak that started in Wuhan was confirmed by the Chinese authorities and later named *coronavirus disease* (COVID-19) by the WHO.^13^ On January 24, 2020, the MOH first advised Israelis to avoid travel to the affected region in China. On January 30, 2020, the WHO announced a global public health concern.^14^ For 32 days, Israel was in a state of anticipatory pre-exposure, without a single confirmed case, but in a state of vigilance. Ongoing COVID-19 updates included a house-quarantine directive issued on February 2, 2020, for travelers returning from known endemic countries. Thirty-two days after the outbreak in China, the first case of SARS-CoV-2 exposure in Israel was identified when a group of South Korean tourists was subsequently known to have been infected. This contact ended the pre-exposure period on February 22, 2020.

During the anticipatory pre-exposure period, MDA acted on several fronts. Although the MOH had not yet issued disease-specific COVID-19 protocols, MDA was receiving calls from individuals displaying febrile or respiratory symptoms, as well as calls from medical clinics, health maintenance organizations, and urgent care centers requesting transport for suspected cases of infection. Dispatchers, aware of the COVID-19 global concern, questioned symptomatic callers about their travel and exposure. At this stage, based on clinical judgment, dispatchers identified relevant callers as “suspected COVID-19.” They then instructed the dispatched teams to use full personal protective gear (gown, N95 mask, gloves) and transport using negative pressure isolation, while informing the receiving hospital of the need for contact precautions. In asymptomatic cases, when the caller expressed concern due to travel and possible exposure, MDA dispatchers advised home-quarantine, as per the general directive of the MOH.

Another front-line role that MDA paramedics and EMTs provided was the border management point. Upon request from the MOH, MDA set up a voluntary COVID-19 checkpoint at Ben-Gurion International Airport in Tel-Aviv. Travelers choosing to be checked for symptoms were directed to the checkpoint where they were examined for fever and questioned regarding breathing difficulties, sore throat, and travel route.

The objective of this descriptive study is to analyze the role of EMS in the pre-exposure period, describing the anticipatory containment and identification efforts implemented before widespread governmental action. These efforts were based on (1) phone diagnosis, dispatch, and transport in case of an anticipatory pandemic outbreak; and (2) border management checkpoints.

## METHODS

The pre-exposure period in Israel was defined as the time from January 21, 2020, when China’s outbreak was confirmed, until February 22, 2020, when the first known case of exposure in Israel was confirmed among a group of South Korean tourists, totaling 32 days.

Calls were identified and categorized by a dispatcher as “suspected COVID-19” based on symptoms (fever over 38°C, breathing difficulties, sore throat) and travel exposure. These calls were divided into 2 categories: (1) calls from individuals identified by an MDA dispatcher as “suspected COVID-19” and (2) calls from medical sources, that is, clinics, HMO physicians, and urgent care centers.

Travelers approaching the voluntary MDA border checkpoint at Ben-Gurion International Airport fell into 4 categories: (1) protective transport to a hospital (return from an endemic area and suspicious symptoms), (2) home-quarantine (return from an endemic area without symptoms), (3) entry refusal (visitor from China, later extended to all of Asia and Italy), and (4) no action required.

This is a descriptive study of MDA activities during the pre-exposure period, including (1) phone diagnosis, dispatch, and transport; and (2) border management checkpoints. De-identified data were extracted from the call management system and the command and control system. All data were entered into Excel (Microsoft, Redmond, WA). The study was approved by the Scientific Committee of Magen David Adom and by the Institutional Review Board of the Shaare Zedek Medical Center (0098-20-SZMC) and received a waiver for the requirement of consent.

## RESULTS

**FIGURE 1:**
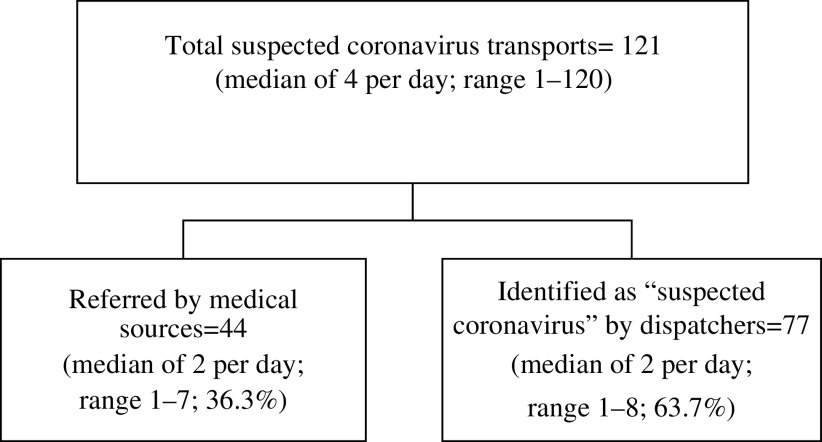
COVID-19 Pre-Exposure Activities of Magen David Adom (From January 21, 2020, Until February 22, 2020).

The total number of protected “suspected COVID-19” transports during the 32 days was 121 (median of 4 per day; range 1–12). Of these, 44 (median of 2 per day; range 1–7; 36.3%) were referred by medical sources, and 77 were identified as “suspected COVID-19” by dispatchers (median of 2 per day; range 1–8; 63.7%) (Figure 1).

**FIGURE 2:**
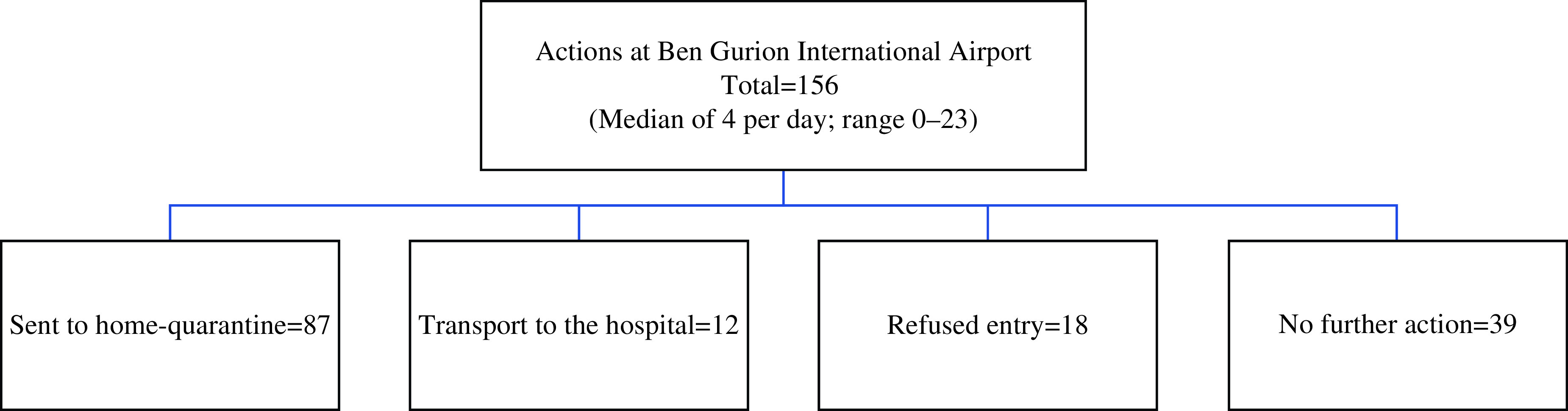
Border Control Actions of Magen David Adom During the Pre-Exposure Period.

The MDA report on the pre-exposure actions at Ben-Gurion International Airport included data from January 31, 2020, until March 1, 2020, several days past the official pre-exposure date. During those dates, 156 people accessed the checkpoint (median of 4 per day; range 0–23) and were categorized into 4 groups: 87 travelers were sent to home-quarantine; 12 received protective transport to the hospital; 18 were refused entry; and 39 required no further action (Figure 2).

## DISCUSSION

The anticipatory pre-exposure period during a global pandemic requires effective management. EMS organizations can take a front-line role in surveillance and containment, helping to postpone infection and minimize exposure to disease. After the official outbreak, MDA acted according to government protocols. In the pre-exposure period, MDA implemented triage based on a merging of standard nonspecific infectious disease protocols and dispatchers’ concern for suspected COVID-19 based on symptoms, travel destinations, and overseas epidemiological exposure. Exercising caution, MDA transported 121 patients during the pre-exposure period who exhibited febrile and respiratory symptoms even when the degree of these symptoms would not have warranted transport in routine cases. At all times, suspected COVID-19 cases were handled with full personal protective equipment, and hospitals were given advanced warning of suspected infection.

As an added attempt at containment pre-exposure, the MOH requested that MDA set up a border control checkpoint for travelers wishing to be checked for COVID-19 symptoms. MDA provided an option that resulted in 156 travelers being checked. It should be noted that, while all visitors from Asia (and afterward Italy) saw signs directing them to the checkpoint, the evaluation itself was voluntary.

The added workload did not require any additional resources from MDA and the routine activities were not affected. During this period, none of the MDA staff tested positive for the virus. In addition, there was no increase in the number of requests for the human resources mental health coordinator.

### Limitations

The design of this study did not allow follow-up of patients and so there is a lack of outcome data. The fact that the airport border checkpoints were voluntary may have enabled visitors and return travelers to avoid detection who theoretically could have been infected. Literature on voluntary testing for viral illness exists in the field of sexually transmitted diseases and HIV.^15-17^ These studies may get large numbers of volunteers as patients who know they have risk factors, are symptomatic, or are positive for the disease and may then get treated with life-saving medicine. On the other hand, a healthy person arriving home or a traveler may be hesitant to get tested at the border or the airport as they may be afraid that they won’t be allowed into the country. Perhaps mandatory testing at the borders should be implemented during this time.

Border checkpoints have many limitations based on the self-identification of travelers and the prevalence of symptoms.^18^ Also, the efficacy of this is questioned as resources may be better applied toward disease control measures in the community.^19^

MDA was not involved in training the airport authority staff in identifying travelers who were suspected to have an infectious disease. Also, no checkpoints were established for the land borders between Egypt, Jordan, and the Palestinian territories. The available information at the time was such that the minimal risk did not justify the large-scale resources needed to staff these checkpoints.

It should be noted that MDA is a national EMS service such that activities related to border management may not be relevant to local pre-hospital services.

## CONCLUSION

Prehospital emergency services can serve as a front-line organization in potential early detection, surveillance, and containment during the anticipatory pre-exposure stage of pandemic outbreaks. EMS can work effectively at border control checkpoints and help contain infection through guidance in instructing home quarantine and providing protected transport. If there will be similar events in the future, public policy-makers should consider establishing *mandatory* checks at *all* border crossings.
